# High-Power Large-Energy Raman Soliton Generations Within a Mode-Locked Yb-Doped Fiber Laser Based on High-Damage-Threshold CVD-MoS_2_ as Modulator

**DOI:** 10.3390/nano9091305

**Published:** 2019-09-12

**Authors:** Pengfei Ma, Wei Lin, Huanian Zhang, Shanhui Xu, Zhongmin Yang

**Affiliations:** 1 State Key Laboratory of Luminescent Materials and Devices, Institute of Optical Communication Materials, South China University of Technology, Guangzhou 510640, Chinaflxshy@scut.edu.cn (S.X.); 2 Shandong Provincial Key Laboratory of Optics and Photonic Devices, School of Physics and Electronics, Shandong Normal University, Jinan 250014, China

**Keywords:** high-damage 2D MoS_2_ materials, ultra-fast optical modulation, mode-locked Raman fiber laser

## Abstract

In our work, based on a high-damage-threshold MoS_2_ saturable absorber (SA), high-power intra-cavity Raman solitons within a passively mode-locked Yb-doped fiber laser were demonstrated successfully for the first time. The damage threshold of the MoS_2_ SA was as high as ~0.48 J/cm^2^. By adjusting the polarization states, stable single- or dual-pulse Raman soliton operations were obtained. The maximum average output power for single-pulse and dual-pulse Raman soliton operations was 80.11 and 89.33 mW, respectively. Our experiment results show significant enhancement in comparison with previous works, which provides fundamental guidance for future designs of high-power, large-energy, intra-cavity Raman soliton generations based on two-dimensional materials as SAs.

## 1. Introduction

Passively mode-locked fiber lasers, as new scientific research and industrial tools, have subversively promoted the scientific research process and industrial development [1,2,3]. In addition, they were always regarded as excellent platforms for soliton investigations [4,5,6,7,8]. The formation of optical solitons within fiber lasers is inseparable from the balance between various non-linear optical effects including self-phase modulation (SPM), cross-phase modulation (XPM), four-wave mixing (FWM), stimulated Brillouin scattering (SBS), stimulated Raman scattering (SRS), and so on [5,6,7,8,9,10]. Studying the mechanism of different non-linear phenomena is of significance in extending the deep understanding of mode-locked fiber lasers. Therefore, as is reported, SRS can expend the spectral range of ion-doped fiber lasers and significantly improve the stability of soliton operations [9,10,11,12,13,14,15]. However, in comparison with widely reported well-known solitons including traditional soliton, dissipative soliton, dark soliton, and so on, Raman solitons were relatively less investigated. Previously, few mode-locked Raman solitons have been obtained within extra-cavity Raman laser demonstrations [9,10,11]. In which, by employing lasers operating at different wavelengths as pump sources, first or high-order stokes Raman solitons were generated within fiber lasers. Besides, intra-cavity mode-locked fiber lasers for the investigations of Raman solitons have also been reported successfully [12,13,14,15]. In 2014, A. F. J. Runge et al. demonstrated an all-normal dispersion mode-locked Raman fiber laser, and they found that SRS could lead to spectral fluctuations [12]. In addition, Kharenko et al. found that SRS enhanced the stability of the mode-locked fiber laser [13]. Bednyakova et al. also proved that the formation of a bound Raman soliton had significance in enhancing the stability of a mode-locked fiber laser [14]. However, in the mentioned works [12,13,14], the SRS effect was always considered as noise, because, in comparison with the intensities of the fundamental wavelengths, the optical intensities of the Raman wavelengths were much lower. Recently, based on a section of highly nonlinear fiber, Zhao et al. demonstrated a passively mode-locked Yb-doped Raman fiber laser; in their work, Raman soliton operation with a maximum average output power of 17 mW, a broad spectral bandwidth of 64 nm, and a high signal-to-noise ratio of 77 dB was obtained [15]. The mentioned experiment results fully proved that the SRS effect has significance in improving the stability of the Raman soliton and broadening the emission spectrum range [12,13,14,15].

In addition, new saturable absorber (SA) materials have profoundly promoted the development of passively mode-locked fiber lasers. So far, novel materials including 2D materials [16,17,18,19,20,21], quantum dots [22,23], metal particles [24,25,26], and so on have been widely employed as SAs for demonstrating soliton generations operating from visible to mid-infrared spectrum bands. Similarly, extra-cavity Raman solitons within new SA-based mode-locked fiber lasers have always been reported by different groups. In 2011, C. E. S. Castellani et al. reported an all-fiber passively mode-locked Raman laser utilizing a broadband carbon nanotubes (CNT)-based SA [27]. In 2012, Zhang et al. demonstrated a linearly polarized 1180 nm passively mode-locked Raman fiber laser using graphene-based SA. Stable nanosecond mode-locked pulses with a maximum average output power of 60 mW at a repetition rate of 0.4 MHz were generated; owing to the extra-cavity demonstration, the optical conversion efficiency was only 0.81% [28]. However, their results confirmed that the combination of the SRS effect and broadband SA devices could offer a prospect of real wavelength-versatile mode-locked laser source adequately [27,28].

So far, to our knowledge, intra-cavity mode-locked Raman solitons based on broadband SA devices have been rarely reported. In comparison with extra-cavity demonstrations, intra-cavity mode-locked fiber laser have the obvious advantages of higher optical conversion efficiency, lower pump threshold, and more compact construction. Thus, based on novel SAs, demonstrating high-power large-energy intra-cavity mode-locked Raman soliton was the major target of our work. Several conditions including high-power pump source, efficient Raman gain coefficient, and high-damage-threshold SAs are necessary for the purpose of generating new SAs-based high-power large-energy intra-cavity mode-locked Raman soliton. On the basis of the considerations above, in detail, in our experiment, two single-mode 976 nm laser diodes (LDs) with a maximum average output power of 900 mW were used for providing enough energy for the Raman conversion. A ~290 m long single mode fiber (SMF) was added into the laser cavity for the enhancement of the Raman gain coefficient. Most importantly, MoS_2_ SA was prepared with a damage threshold higher than 0.48 J/cm^2^ based on the chemical vapor deposition (CVD) technique. The reasons for the selection of MoS_2_ as the SA substrate can be stated as below: firstly, MoS_2_ has been widely employed as SAs and proved to exhibit excellent nonlinear optical properties including wide absorption band, suitable bandgap value, ultra-fast recovery time, high damage threshold, and so on. In addition, large-scale MoS_2_ film with flat surface characteristics and controllable layered structure, which have significance in enhancing the damage threshold of the materials, can be prepared by the CVD technique maturely.

In this work, based on the mentioned combination of high-power pump source, efficient Raman gain coefficient, and high-damage-threshold SA, high-power and large energy Raman solitons have been obtained successfully. Single-pulse Raman soliton operation with a maximum average output power of 80.11 mW at a pulse repetition rate of 683.5 kHz was generated under the pump power of 1620 mW, corresponding to a pulse energy of 117.4 nJ and an optical conversion efficiency of 4.93%. In addition, by controlling the polarization states within the laser cavity, dual-pulse Raman soliton operation with a maximum average output power of 89.33 mW was also obtained. In comparison with previous works, our experiment results show significant enhancement. The results fully prove the superiority of our experimental design scheme, and provide fundamental guidance for future designs of high-power, large-energy, mode-locked Raman soliton fiber lasers based on two-dimensional materials as SAs.

## 2. Fabrication and Characterization of the MoS_2_ Modulator

Previously, fabrication techniques including mechanical exfoliation (ME), liquid exfoliation (LE) or liquid phase exfoliation (LPE), pulsed laser deposition (PLD), plasma-assisted fabrication, chemical vapor deposition (CVD), and so on have been widely employed for preparing layered 2D materials appropriately [29,30,31,32,33]. Thereinto, CVD was regarded as an efficient method for the production of single- and few-layer 2D materials with controllable layers and uniform shape [29,30,31]. Especially, CVD-2D materials also exhibit smooth surface, which is beneficial for improving the damage threshold of the materials. In our work, we employed the CVD method to prepare MoS_2_ materials on the substrate of fluorophlogopite mica (FM) and sapphire substrate, respectively. MoS_2_ deposited on FM can be easily peeled off from the substrate and act as the SA. In addition, MoS_2_ deposited on sapphire substrate was employed for the purpose of morphology characterization.

**Figure 1:**
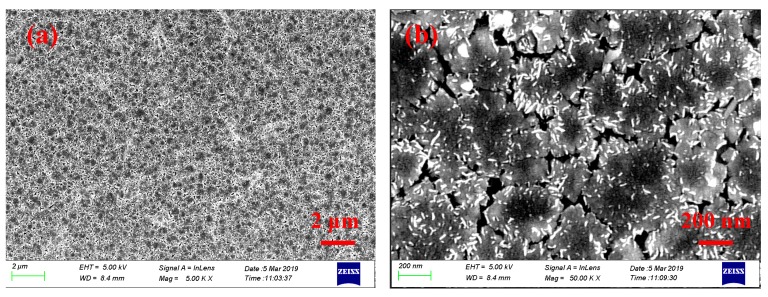
Scanning electrical microscope (SEM) images of the prepared MoS_2_ film recorded under different resolutions. (**a**) Under a resolution of 2 μm. (**b**) Under a resolution of 200 nm.

Figure 1a,b show the scanning electrical microscope (SEM) images of the prepared MoS_2_, which were recorded under different resolutions by a scanning electrical microscope (Sigma 500, ZEISS, Zeiss, Jena, Germany). As is depicted in Figure 1a, relatively continuous large-scale MoS_2_ film was prepared. In addition, the obvious smooth surface characteristic of the prepared MoS_2_ is shown in Figure 1b, large-scale materials with smooth surface were beneficial for the improvement of the damage threshold of the SA; thus, based on the prepared MoS_2_ film, SA with a high damage threshold can be expected.

**Figure 2:**
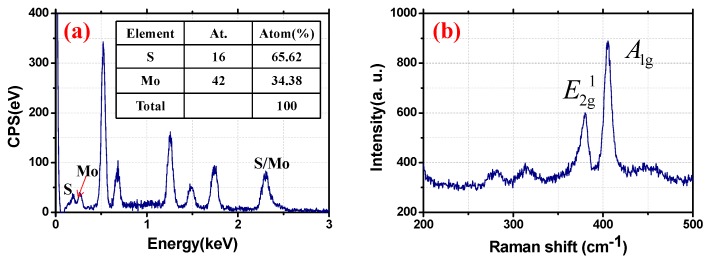
(**a**) The energy-dispersive X-ray (EDX) spectroscopy of the MoS_2_; (**b**) the Raman spectrum of the MoS_2_.

The energy-dispersive X-ray (EDX) spectrum recorded by an energy dispersive spectrometer (EDS, XFlash 6130, Bruker, Karlsruhe, Germany) with obvious peaks associated with Mo and S is shown in Figure 2a. The measured atomic ratio between Mo and S is about 34:66, which is compatible with the chemical formula of MoS_2_. In addition, CPS is the count per second. On the basis of a Raman spectroscopy (Horiba HR Evolution), the Raman spectrum with Raman shifts corresponding to the typical out-of-plane A_1g_ (~405.493 cm^−1^) and the in-plane E_2g_^1^ (~379.978 cm^−1^) modes of MoS_2_ was also recorded and shown in Figure 2b; the recorded Raman shifts are in good agreement with the previous works [34,35]. The EDX and Raman results represent obvious evidence proving that the pure MoS_2_ film was prepared in our work.

**Figure 3:**
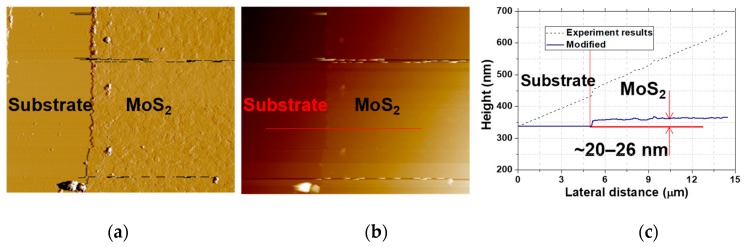
(**a,b**) The atomic force microscope (AFM) images of the MoS_2_ under different resolutions; (**c**) the corresponding thicknesses characteristics.

In our experiment, we employed an atomic force microscope (AFM, Bruker Multimode 8, Bruker, Karlsruhe, Germany), and layered properties of the MoS_2_ deposited on sapphire substrate were also tested for better understanding the layer-dependent nonlinear absorption properties such as modulation depth, saturable intensity, and so on. Figure 3a,b show the recorded AFM images of the MoS_2_; a large-area MoS_2_ film with flat surface was fabricated successfully. Figure 3c shows the corresponding thicknesses of the marked areas of Figure 3b. Firstly, the measured results proved that the substrate exhibited an obvious inclination angle, thus the measured results were modified by reducing the effect of the slope efficiency; also shown in Figure 3c. As is shown, the thicknesses of the marked area are about 20–26 nm, corresponding to the number of layers of about 30–40 [34]. The overall results reveal that MoS_2_ materials with a smooth surface and uniform layer numbers were fabricated successfully. Besides, as depicted in Figure 1, the MoS_2_ film with thickness around 20 nm appears to be non-continuous; however, because of the limitation of the used resolution of the AFM, the characteristics of the non-continuous surface were not investigated. In our future work, however, we will try to study the non-continuous characteristics of the MoS_2_ and its potential influence on the properties of the SA.

**Figure 4:**
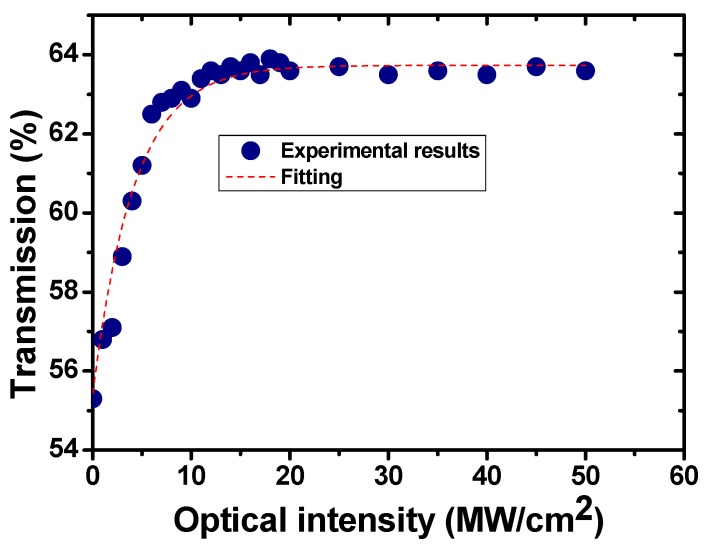
Power-dependent nonlinear absorption property of the MoS_2_ SA.

The nonlinear optical properties of the prepared MoS_2_ SA were tested based on a well-known two-arm detection method [31,32,33]. In our work, a home-made Yb-doped mode-locked fiber laser operating at the central wavelength of 1060.36 nm with a 3 dB spectral width of 1.63 nm, a pulse width of 12.6 ps, a maximum average output power of 30 mW, and a pulse repetition rate of 13 MHz was employed as the pump source for the testing. The measured results are provided in Figure 4. For a traditional 2D material, its nonlinear optical properties, including the saturation intensity, modulation depth, and so on, could be calculated by fitting the measured results based on the widely-used formula [31,32,33]:

(1) $$T(I)=1-T_{ns}-\Delta T\times \exp(-I/I_{sat}),$$

where *T*, *T_ns_*, *ΔT*, *I*, and *I_sat_* are transmission, non-saturable absorbance, modulation depth, input intensity of laser, and saturation intensity, respectively. Finally, the saturation intensity and modulation depth of the prepared MoS_2_ SA are calculated to be 4.2 MW/cm^2^ and 8.3%, respectively. Additionally, for a 2D material, its nonlinear optical properties can also be investigated by fitting the date using the method explained in the work of [32]. Thus, the saturation power and modulation depth were converted to be 0.44 mW and 8.3%, respectively.

In general, based on the CVD method, the prepared film may contain MoS_2_ crystallites with many different orientations. However, the relationship between the mentioned tested properties of the MoS_2_ and the orientation of MoS_2_ is not yet explicit. Thus, the phenomenon presented in our work may not applicable to other kinds of flat MoS_2_ crystals such as flakes, CVD triangular domains, and so on.

## 3. Experimental Setup

**Figure 5:**
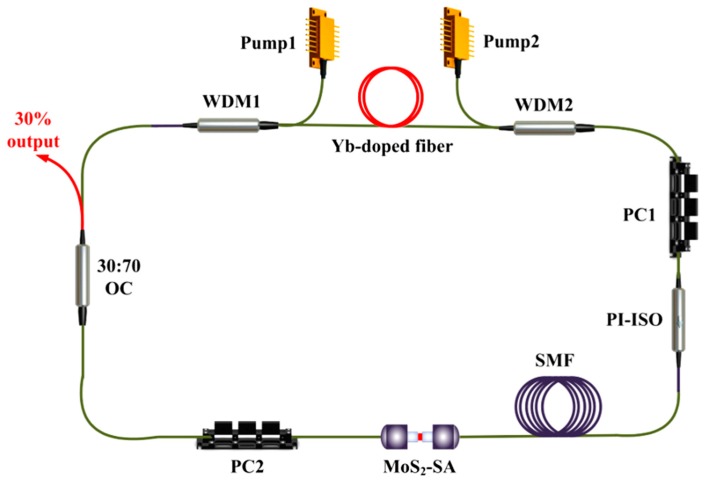
Experimental setup of the Yb-doped Raman soliton mode-locked fiber laser. PI-ISO, polarization independent isolator; PC, polarization controller; SMF, single mode fiber; WDM, wavelength division multiplexer; OC, output coupler.

The experimental setup for the generations of Raman solitons is shown in Figure 5. As is depicted, a double-end-pumped ring laser cavity is designed. Two 976 nm laser diodes (LDs) with a maximum average output power of 900 mW are used as pump sources for providing enough energy for the Raman conversion, which are guided into the laser cavity through two 980/1030 wavelength division multiplexers (WDM1 and WDM2). A piece of ~0.9 m long Yb-doped fiber (Nufern, LMA-YSF-10/125, Connecticut, USA) is used as the laser gain medium. A polarization independent isolator (PI-ISO) and two polarization controllers (PC1 and PC2) are used to guarantee the unidirectional transmission and adjust the polarization states in the laser cavity. The SA is set between the PC2 and PI-ISO. The output fiber laser is output from the 30% port of a 30:70 output coupler (OC). Additionally, a piece of ~290 m SMF is added into the ring laser cavity for enhancing the Raman gain coefficient. Finally, the total cavity and the overall length of the passive fiber is about ~300.63 m.

## 4. Results and Discussion

Optimization of the Raman gain coefficient within the laser cavity is essential for demonstrating mode-locked Raman solitons. Thus, in our work, the first step is to optimize the length of the SMF to achieve the Raman soliton operation. When the length of the laser cavity was longer than ~300 m, Raman soliton can be obtained regularly by adjusting the pump power and the state of the PCs. Although a longer cavity length corresponds to a larger Raman gain coefficient and low threshold power for the Raman soliton generation, a longer length also leads to the decrease of the pulse energy owing to the pulse split caused by the effect of self-phase modulation (SPM), cross-phase modulation (XPM), and so on [5,6,7]. Accordingly, the final length of the cavity was selected to be ~300 m. Additionally, because of the fact that the obvious Kerr effect caused by high pump power within long-length SMF will lead to the formation of self-mode-locked or Q-switched operations, it is necessary to confirm whether there is a self-mode-locked phenomenon occurring in the experiment. Thus, firstly, the SA was removed from the laser cavity, by adjusting the pump power and the states of the PCs, no mode-locked pulses were recorded, indicating that the SA was fully responsible for the modulation effect. In the experiment, by adjusting the pump power and the states of the PCs, Raman solitons with different pulse shapes were obtained successfully, and are discussed separately below.

### 4.1. Single-Pulse Raman Soliton

**Figure 6:**
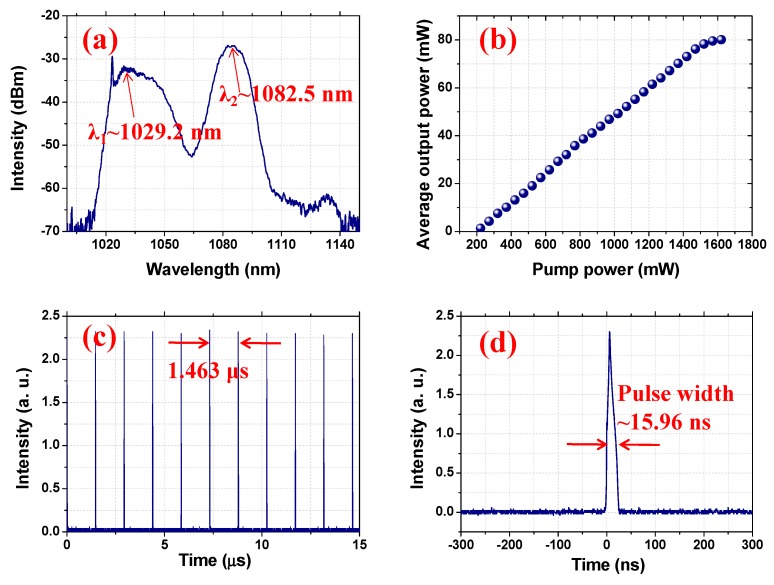
The typical characteristics of the single-pulse mode-locked fiber laser under the maximum pump power: (**a**) optical spectrum; (**b**) average output power scaling as the increase of pump power; (**c**) pulse train; (**d**) typical single-pulse.

By adjusting state of the PCs, single-pulse Raman soliton operations can be recorded with the pump power ranging from 220 to 1620 mW. The output characteristics of the single-pulse Raman soliton under the maximum pump power of 1620 mW are shown in Figure 6. In detail, Figure 6a shows the emission spectrum recorded with the resolution of 0.05 nm; two spectrum peaks with central wavelengths of 1029.2 and 1082.5 nm, which correspond to the fundamental and first Stokes Raman wavelengths, are described. The emission spectrum fully proves the formation of the Raman soliton. The relationship between the average output power and the pump power is shown in Figure 6b. The maximum average output power is as high as 80.11 mW under a pump power of 1620 mW, corresponding to an optical conversion efficiency of 4.95%. In addition, the results show that the output power increases with the increase of the pump power at low pump intensity. However, when the pump power is higher than 1520 mW, the output power has a tendency to saturate. The typical recorded pulse train of the Raman soliton operation is depicted in Figure 6c; the measured pulse-to-pulse time is 1.463 μs, corresponding to a cavity-length matched frequency of 683.5 kHz. The single-pulse shape with a pulse width of 15.96 ns is recorded and shown in Figure 6d. Thus, the corresponding largest pulse energy and highest peak power were 117.4 nJ and 7.35 W, respectively.

**Figure 7:**
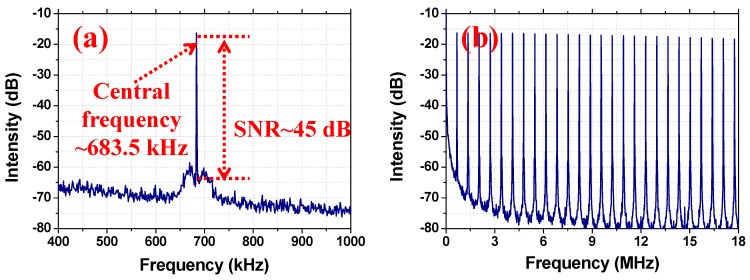
The operation frequency information of the single-pulse mode-locked Raman soliton operation. (**a**) Recorded within 600 kHz bandwidth. (**b**) Recorded within 18 MHz bandwidth. SNR, signal-to-noise ratio.

The operation frequency (OF) spectra of the mode-locked Raman soliton operations recorded under different band widths and resolutions are depicted in Figure 7. The fundamental frequency with a signal-to-background ratio of ~45 dB is located at 683.5 kHz. In addition, the OF spectrum recorded within an 18 MHz spectrum bandwidth also shows a large signal-to-noise ratio (SNR), which provides obvious evidence of a stable mode-locked state.

**Figure 8:**
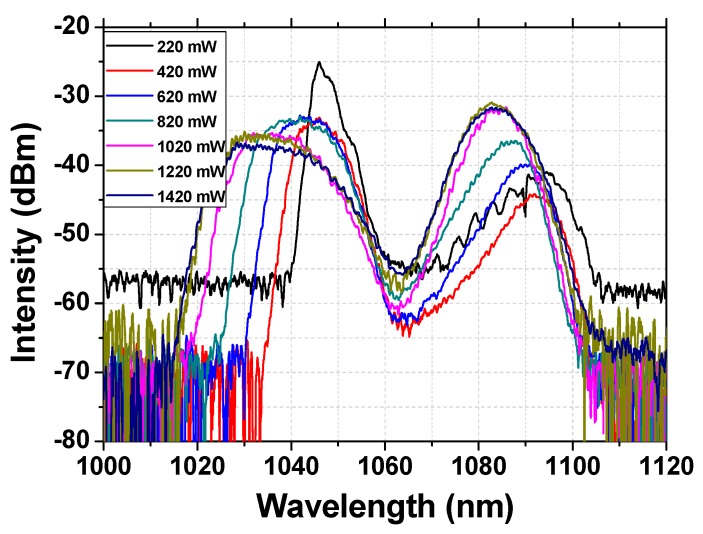
Emission spectra of the single-pulse mode-locked laser under different pump powers.

The evolution of the emission spectra recorded under different pump power is shown in Figure 8, which will be beneficial for understanding the formation of the Raman soliton. Firstly, under lower pump power (<820 mW), the intensity of the Raman wavelength is much lower than that of the fundamental wavelength. However, with the increasing of the pump power, the intensity of the Raman soliton exhibits obvious enhancement; meanwhile, owing to the energy transfer, the intensity of the fundamental frequency decreases gradually, until the end, and the Raman intensity exceeds the intensity of the fundamental frequency. The described evidence fully presents the formation of the Raman solitons.

### 4.2. Dual-Pulse Raman Soliton

**Figure 9:**
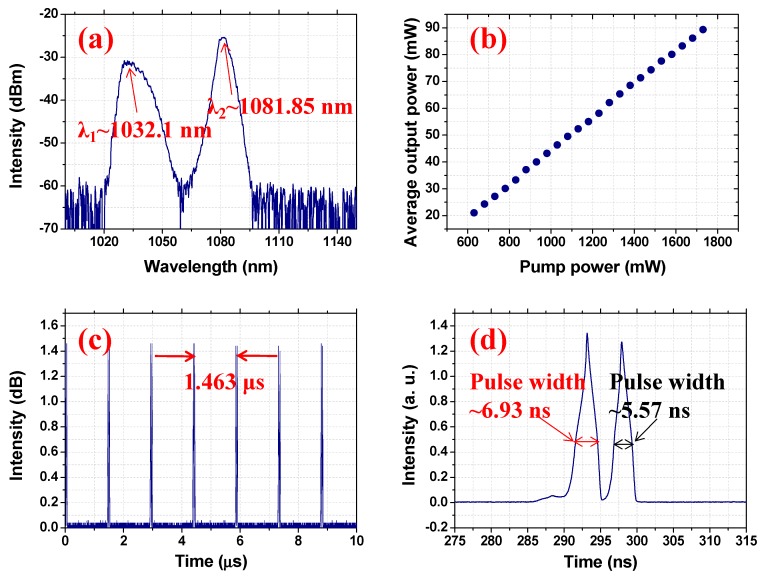
The typical characteristics of the dual-pulse mode-locked fiber laser under the maximum pump power: (**a**) optical spectrum; (**b**) average output power scaling as the increase of pump power; (**c**) pulse train; (**d**) typical single-pulse.

Meanwhile, when the pump power is higher than 630 mW, the adjustment of the state of PCs also leads to the formation of dual-pulse Raman soliton. The recorded output performances under the available maximum pump power of 1730 mW are shown in Figure 9. Figure 9a shows the emission spectrum, which exhibits two obvious peaks corresponding to the first Stokes Raman and the fundamental wavelength. The central wavelengths of the fundamental and first Stokes Raman laser are 1032.1 and 1081.85 nm, respectively. The characteristics of the average output power are shown in Figure 9b; the maximum average output power is 89.33 mW under the pump power of 1730 mW. However, no saturated tendency is recorded with the increase of the pump power, indicating that higher average output power can be expected under high pump power, which is different from the mentioned single-pulse operation. However, because of the limitation of the available maximum pump power, the output performance under higher pump powers is not investigated. The recorded pulse train and typical pulse shape are depicted in Figure 9c,d; the pulse repetition rate was also 683.5 kHz, corresponding to a pulse energy of as high as 130.7 nJ. However, as shown in Figure 9d, the pulse shape splits into two pulses, and the corresponding pulse widths are 6.93 and 5.57 ns. Additionally, as it is mentioned that a 30:70 OC is used for outputting the pulse energy through its 30% port, the corresponding pulse energy within the laser cavity is calculated to be about 305 nJ, indicating that the damage threshold of the SA is higher than 0.48 J/cm^2^, which exhibits significant enhancement.

**Figure 10:**
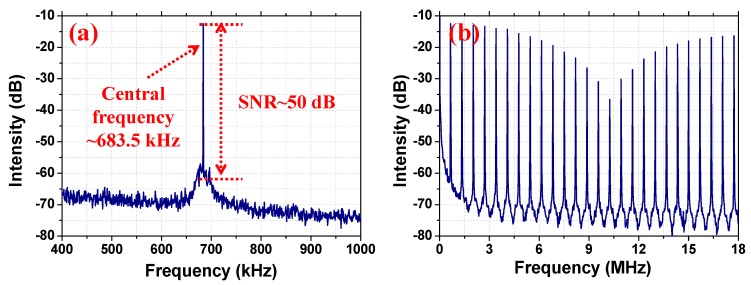
The operation frequency information of the dual-pulse mode-locked Raman soliton operation. (**a**) Recorded within 600 kHz bandwidth. (**b**) Recorded within 18 MHz bandwidth.

The recorded OF spectra of the dual-pulse Raman soliton operation are deposited in Figure 10. The signal-to-background ratio at the fundamental frequency of 683.5 kHz is as high as 50 dB, which is larger than that of the single-pulse mode-locked operation, indicating that the dual-pulse operation exhibits a more stable state than the single pulse Raman soliton operation. The OF spectrum recorded within an 18 MHz spectrum bandwidth also shows a large signal-to-noise ratio, and the stability of dual-pulse mode-locked Raman soliton fiber laser operation is further proved.

**Figure 11:**
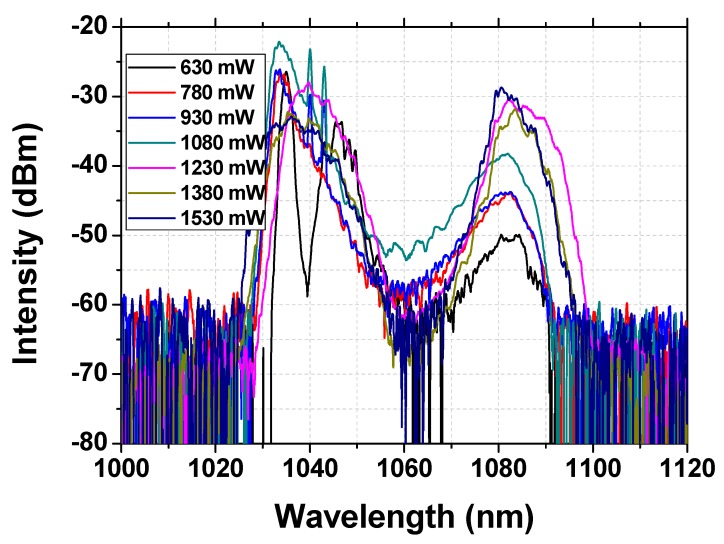
Emission spectra of the dual-pulse mode-locked laser under different pump powers.

The spectral evolution of the dual-pulse Raman soliton operation was also recorded and is shown in Figure 11. As in the single-pulse case, with the increasing of the pump power, the intensity of Raman solitons increases gradually. Meanwhile, the intensity of the fundamental frequency light gradually decreases as a result of the energy transfer. Finally, the intensity of the Raman soliton exceeds that of the fundamental frequency. The evolution of the spectrum also fully shows the formation and enhancement of Raman solitons.

**Figure 12:**
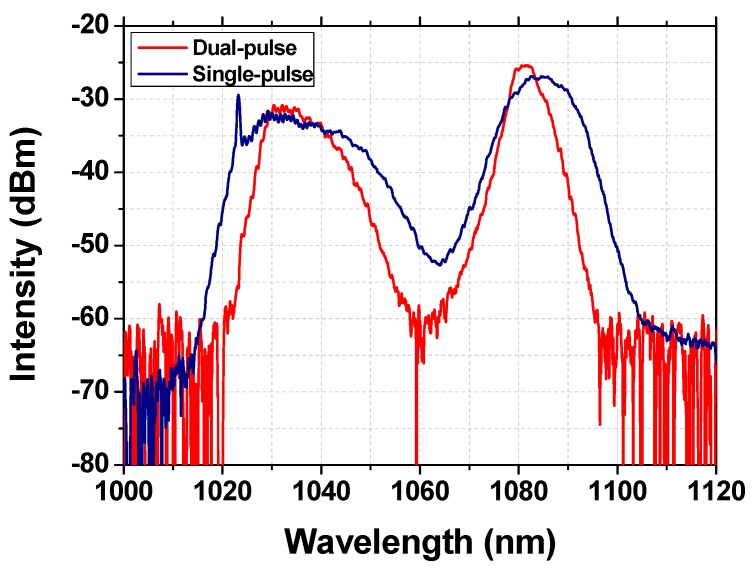
Comparison of the emission spectra of the single- and dual-pulse mode-locked lasers under maximum pump powers.

For understanding the differences between the pulse shapes of the two mentioned kinds of Raman solitons, the comparison of the corresponding emission spectra under the maximum pump power is depicted in Figure 12. As is shown, for the single pulse Raman soliton, the emission spectrum exhibits a wider 3 dB spectrum width for both the fundamental and first Stokes wavelengths. However, the emission spectrum of the dual-pulse Raman soliton exhibits relatively more independent emission peaks, which will lead to a dual-pulse operation.

In Table 1, typical output characteristics of extra-cavity and intra-cavity mode-locked Raman soliton operations are compared. Firstly, owing to the employment of an intra-cavity demonstration, the optical-to-optical conversion efficiencies of our work are much higher than those of the extra-cavity reports, which is beneficial to the higher optical intensity within the laser cavity. Secondly, profiting from the use of the high power pump source and high-damage-threshold MoS_2_ SA, the average output power of our work is much higher than the previous results obtained within extra-cavity and intra-cavity demonstrations. In conclusion, the excellent output characteristics prove that our design has great advantages in generating Raman soliton generations with high average output power and large energy. In addition, the method for preparing saturable absorbers used in our work also provides an effective reference for promoting the application of two-dimensional materials in the field of ultrafast optoelectronic devices, as well as for studying the influence of non-linear parameters such as absorption and transmittance of materials on demonstrating new saturable absorbers [36,37,38].

## 5. Conclusions

In conclusion, based on the CVD method, MoS_2_ SA with a damage threshold of higher than 0.48 J/cm^2^ was prepared and employed for generating high-power, large-energy, mode-locked, intra-cavity Raman solitons within a Yb-doped fiber laser. Raman soliton operations with different pulse shapes were obtained successfully. The maximum average output powers were 80.11 and 89.33 mW, respectively. In addition, this was the first demonstration in which an intra-cavity mode-locked Raman laser based on 2D materials was reported. Our experimental design and results open a new avenue for generating high-power, large-energy, mode-locked Raman soliton fiber lasers based on two-dimensional materials as SAs.

## Figures and Tables

**Table 1:** Comparison of Raman soliton mode-locked fiber lasers.

Cavity Type	*λ_F_/λ_R_* (nm/nm)	*P_ave_*/mW	*η_opo_*/%	*f*/MHz	*RF*/dB	*E_pulse_*/ nJ	Ref
Extra-cavity	1555/1666	0.08	~	1.72	~35	0.047	27
Extra-cavity	1120/1180	60	0.81	0.4	56	150	28
Intra-cavity	1040.16/1086.31	17	4.78	19.6065	77	0.87	15
Intra-cavity	1029.20/1085.85	80.11	4.95	0.6835	~45	117.4	Our
Intra-cavity	1032.95/1081.85	89.33	5.16	0.6835	~50	130.7	Our

*λ_F_*: fundamental wavelength; *λ_R_*: first Stokes Raman wavelength; *P_ave_*: average output power, *η*: optical-to-optical conversion efficiency; *f*: pulse repetition rate; *RF*: radio frequency; *E_pulse_*: pulse energy.

## References

[1] Schliesser A., Picqué N., Hänsch T.W. (2012). Mid-infrared frequency combs. Nat. Photon..

[2] Udem T., Holzwarth R., Hänsch T.W. (2002). Optical frequency metrology. Nature.

[3] Fermann M.E., Hartl I. (2013). Ultrafast fibre lasers. Nat. Photon..

[4] Martinez A., Sun Z. (2013). Nanotube and graphene saturable absorbers for fibre lasers. Nat. Photon..

[5] Grelu P., Akhmediev N. (2012). Dissipative solitons for mode-locked lasers. Nat. Photon..

[6] Sun Z., Hasan T., Torrisi F., Popa D., Privitera G., Wang F., Bonaccorso F., Basko D.M., Ferrari A.C. (2010). Graphene mode-locked ultrafast laser. ACS Nano.

[7] Oktem B., Ülgüdür C., Ilday F.Ö. (2010). Soliton-similariton fibre laser. Nat. Photon..

[8] Lecaplain C., Grelu P., Soto-Crespo J.M., Akhmediev N. (2012). Dissipative rogue waves generated by chaotic pulse bunching in a mode-locked laser. Phys. Rev. Lett..

[9] Schröder J., Coen S., Vanholsbeeck F., Sylvestre T. (2006). Passively mode-locked Raman fiber laser with 100 GHz repetition rate. Opt. Lett..

[10] Chestnut D.A., Taylor J.R. (2005). Wavelength-versatile subpicosecond pulsed lasers using Raman gain in figure-of eight fiber geometries. Opt. Lett..

[11] Chamorovskiy A., Rantamäki A., Sirbu A., Mereuta A., Kapon E., Okhotnikov O.G. (2010). 1.38-µm mode-locked Raman fiber laser pumped by semiconductor disk laser. Opt. Express.

[12] Runge A.F., Aguergaray C., Broderick N.G., Erkintalo M. (2014). Raman rogue waves in a partially mode-locked fiber. Opt. Lett..

[13] Kharenko D.S., Podivilov E.V., Apolonski A.A., Babin S.A. (2012). 20 nJ 200 fs all-fiber highly chirped dissipative soliton oscillator. Opt. Lett..

[14] Bednyakova A.E., Babin S.A., Kharenko D.S., Podivilov E.V., Fedoruk M.P., Kalashnikov V.L., Apolonski A. (2013). Evolution of dissipative solitons in a fiber laser oscillator in the presence of strong Raman scattering. Opt. Express.

[15] Zhao L., Yao P.J., Gu C., Xu L.X. (2018). Raman-assisted passively mode-locked fiber laser. Chin. Phys. Lett..

[16] Ma P.F., Lin W., Zhang H.N., Xu S.H., Yang Z.M. (2019). Nonlinear absorption properties of Cr_2_Ge_2_Te_6_ and its application as an ultra-fast optical modulator. Nanomaterials.

[17] Choi S.Y., Cho D.K., Song Y.W., Oh K., Kim K., Rotermund F., Yeom D.I. (2012). Graphene-filled hollow optical fiber saturable absorber for efficient soliton fiber laser mode locking. Opt. Express.

[18] Niu K.D., Sun R.Y., Chen Q.Y., Man B.Y., Zhang H.N. (2018). Passively mode-locked Er-doped fiber laser based on SnS_2_ nanosheets as a saturable absorber. Photon. Res..

[19] Liu H., Luo A.P., Wang F.Z., Tang R., Liu M., Luo Z.C., Xu W.C., Zhao C.J., Zhang H. (2014). Femtosecond pulse erbium-doped fiber laser by a few-layer MoS_2_ saturable absorber. Opt. Lett..

[20] Xu N.N., Ming N., Han X.L., Man B.Y., Zhang H. (2019). Large-energy passively Q-switched Er-doped fiber laser based on CVD-Bi_2_Se_3_ as saturable absorber. Opt. Mater. Express.

[21] Jhon Y.I., Koo J., Anasori B., Seo M., Lee J.H., Gogotsi Y., Jhon Y.M. (2017). Metallic MXene saturable absorber for femtosecond mode-locked lasers. Adv. Mater..

[22] Ming N., Tao S.N., Yang W.Q., Chen Q.Y., Sun R.Y., Wang C., Wang S.Y., Man B.Y., Zhang H.N. (2018). Mode-locked Er-doped fiber laser based on PbS/CdS core/shell quantum dots as saturable absorber. Opt. Express.

[23] Shi Y.H., Long H., Liu S.X., Tsang Y.H., Wen Q. (2018). Ultrasmall 2D NbSe_2_ based quantum dots used for low threshold ultrafast lasers. J. Mater. Chem. C.

[24] Kang Z., Liu M.Y., Li Z.W., Li S.Q., Jia Z.X., Liu C.Z., Qin W.P., Qin G.S. (2018). Passively Q-switched erbium doped fiber laser using a gold nanostars based saturable absorber. Photon. Res..

[25] Zhang H.N., Liu J. (2016). Gold nanobipyramids as saturable absorbers for passively Q-switched laser generation in the 1.1 μm region. Opt. Lett..

[26] Kang Z., Liu M.Y., Gao X.J., Li N., Yin S.Y., Qin G.S., Qin W.P. (2015). Mode-locked thulium-doped fiber laser at 1982 nm by using a gold nanorods saturable absorber. Laser Phys. Lett..

[27] Castellani C.E.S., Kelleher E.J.R., Travers J.C., Popa D., Hasan T., Sun Z., Flahaut E., Ferrari A.C., Popov S.V., Taylor J.R. (2011). Ultrafast Raman laser mode-locked by nanotubes. Opt. Lett..

[28] Zhang L., Wang G.Z., Hu J.M., Wang J.H., Fan J.T., Wang J., Feng Y. (2012). Linearly polarized 1180-nm Raman fiber laser mode locked by graphene. IEEE Photon. J..

[29] Dhanabalan S.C., Ponraj J.S., Guo Z.N., Li S.J., Bao Q.L., Zhang H. (2017). Emerging trends in phosphorene fabrication towards next generation devices. Adv. Sci..

[30] He J.S., Tao L.L., Zhang H., Zhou B., Li J.B. (2019). Emerging 2D materials beyond graphene for ultrashort pulse generation in fiber lasers. Nanoscale.

[31] Guo B. (2018). 2D noncarbon materials-based nonlinear optical devices for ultrafast photonics. Chin. Opt. Lett..

[32] Du J., Wang Q.K., Jiang G.B., Xu C.W., Zhao C.J., Xiang Y.J., Chen Y., Wen S.C., Zhang H. (2014). Ytterbium-doped fiber laser passively mode locked by few-layer Molybdenum Disulfide (MoS_2_) saturable absorber functioned with evanescent field interaction. Sci. Rep..

[33] Hu Q.Y., Zhang X.Y., Liu Z.J., Li P., Li M., Cong Z.H., Qin Z.G., Chen X.H. (2019). High-order harmonic mode-locked Yb-doped fiber laser based on a SnSe_2_ saturable absorber. Opt. Laser Technol..

[34] Zhan Y.J., Liu Z., Najmaei S., Ajayan P.M., Lou J. (2012). Large-area vapor-phase growth and characterization of MoS_2_ atomic layers on a SiO_2_ substrate. Small.

[35] Nguyen T.P., Sohn W., Oh J.H., Jang H.W., Kim S.Y. (2016). Size-dependent properties of two-dimensional MoS_2_ and WS_2_. J. Phys. Chem. C.

[36] Bao Q.L., Zhang H., Wang Y., Ni Z.H., Yan Y.L., Shen Z.X., Loh K.P., Tang D.Y. (2009). Atomic-layer graphene as a saturable absorber for ultrafast pulsed lasers. Adv. Funct. Mater..

[37] Marchena M., Song Z., Senaratne W., Li C., Liu X.Y., Baker D., Ferrer J.C., Mazumder P., Soni K., Lee R. (2017). Direct growth of 2D and 3D graphene nano-structures over large glass substrates by tuning a sacrificial Cu-template layer. 2D Mater..

[38] Traynor N.J., Grudinin A.B., Pruneri V., Sysoliatin A.A., Semenov V.A. (1997). Tunable source of picosecond pulses around 1550 nm for all-optical processing. Opt. Commun..

